# Sarilumab versus standard of care for the early treatment of COVID-19 pneumonia in hospitalized patients: SARTRE: a structured summary of a study protocol for a randomised controlled trial

**DOI:** 10.1186/s13063-020-04633-3

**Published:** 2020-09-16

**Authors:** Antonio F. Caballero Bermejo, Belén Ruiz-Antorán, Ana Fernández Cruz, Elena Diago Sempere, Alejandro Callejas Díaz, Elena Múñez Rubio, Cristina Avendaño-Solá, Antonio Ramos Martínez, Aránzazu Sancho López, Puerta de Hierro COVID-19 Study Group

**Affiliations:** 1 Clinical Pharmacology Department, Hospital Universitario Puerta de Hierro-Majadahonda, Instituto de Investigación Sanitaria Puerta de Hierro - Segovia de Arana, Madrid, Spain; 2 Infectious Diseases Unit, Internal Medicine Department, Hospital Universitario Puerta de Hierro-Majadahonda, Instituto de Investigación Sanitaria Puerta de Hierro - Segovia de Arana, Madrid, Spain

**Keywords:** COVID-19, SARS-CoV-2, Randomised controlled trial, protocol, sarilumab, anti-IL6 inhibitor, severe interstitial pneumonia, corticosteroids, early intervention

## Abstract

**Objectives:**

In some patients, acute, life-threatening respiratory injury produced by viruses such as SARS-CoV and other viral pneumonia are associated with an over-exuberant cytokine release. Elevated levels of blood IL-6 had been identified as a one of the risk factors associated with severe COVID-19 disease.

Anti-IL6 inhibitors are among the therapeutic armamentarium for preventing the fatal consequences of acute respiratory and multi organ failure in around 20% of the COVID-19 infected patients. At present, their use is prioritized to patients with severe interstitial pneumonia (Brescia-COVID Scale-COVID 2-3) with hyperinflammation as determined by the presence of elevated IL6 and/or d-dimer, or progressive d-dimer increase, in patients who otherwise are subsidiary to ICU admission. However, many uncertainties remain on the actual role of anti-IL6 inhibitors in this setting, and whether current use and timing is the right one. There is the hypothesis that the use of anti-IL6 inhibitors at an earlier state during the hyperinflammatory syndrome would be beneficial and may avoid progressing to ARDS. On the other hand, the standard of care has changed and nowadays the use of corticosteroids has become part of the SOC in the treatment of COVID-19 pneumonia. Our limited experience suggests that better treatment outcomes can be achieved when combining IL6-inhibitors (e.g. sarilumab) with corticosteroids.

The aim of the present study is to evaluate if an earlier therapeutic intervention with sarilumab plus SOC (including corticosteroids) may be more effective than current standard of care alone, in preventing progression to respiratory failure in COVID-19 infected patients with interstitial pneumonia. This study will also provide supportive evidence to that provided by currently ongoing studies on the efficacy and safety of sarilumab in this clinical context.

**Trial design:**

A phase two multi-center randomised controlled trial (RCT) with two parallel arms (1:1 ratio).

**Participants:**

They will be hospitalized patients, of at least 18 years of age, with severe COVID-19 who have positive RT-PCR test and have radiographic evidence of pulmonary infiltrates by imaging or rales/crackles on exam and SpO2 ≤ 94% on room air that requires supplemental oxygen. Patients must present elevation of inflammatory parameters (IL-6 > 40 pg/mL or d-dimer >1.0 mcg/ml) or, alternatively, progressive worsening in at least two of these inflammatory parameters in the prior 24-48h: CRP, LDH, serum ferritin, lymphopenia, or d-dimer.

Exclusion criteria: high oxygen requirements (including face mask with reservoir, non-invasive mechanical ventilation or high flow nasal cannula, or mechanical ventilation), admission to ICU, pregnancy or lactation, allergy or hypersensitivity to sarilumab or corticoesteroids, immunosuppressive antibody therapy within the past 5 months, AST/ALT values > 10 x ULN, neutropenia (< 0.5 x 109/L), severe thrombocytopenia (< 50 x 109/L), sepsis caused by an alternative pathogen, diverticulitis with risk of perforation or ongoing infectious dermatitis.

The study will be conducted in several hospitals in Spain.

**Intervention and comparator:**

Patients randomised to the experimental arm will receive sarilumab + methylprednisolone plus SOC for COVID-19. Patients included in the control arm will receive methylprednisolone plus SOC for COVID-19. Corticosteroids will be given to all patients at a 1mg/kg/d of methylprednisolone for at least 3 days. Clinical follow-up visits will be performed at 3, 5, and 15 days after treatment randomization.

Patients in the control group (SOC group without sarilumab) progressing to Brescia- COVID 2-3 plus inflammatory markers, will be given the option to be rescued with sarilumab at the same doses and, in that case, be included in an open-label phase and be followed up for additional weeks (with visits at 3, 7 and 15 days after sarilumab rescue administration). Patients randomly assigned to sarilumab therapy at baseline progressing to Brescia-COVID 2-3 will be rescued according to local clinical practice protocols.

A final follow-up visit will be conducted for all patients at day 29 from randomization, regardless of initial treatment assignment.

**Main outcomes:**

Primary end point is the proportion of patients progressing to either severe respiratory failure (Brescia-COVID ≥2), ICU admission, or death.

**Randomization:**

Randomization codes were produced by means of the PROC PLAN of the SAS system, with a 1:1 assignment ratio, stratifying by centre and using blocks multiple of 2 elements. The randomization schedule will be managed through the eCRF in a concealed manner.

**Blinding (masking):**

All study drugs will be administered as open label. No blinding methods will be used in this trial.

**Numbers to be randomised (simple size):**

The target sample size will be 200 COVID-19 patients, who will be allocated randomly to control arm (100) and treatment arm (100).

**Trial status:**

Protocol Code: SARTRE Protocol Date: May 05th 2020. Version: 2.0

The study has been approved by the Spanish Competent Authority (AEMPS) as a low intervention clinical trial.

Start of recruitment: August, 2020

End of recruitment: May, 2021

**Trial registration:**

Identifier: EudraCT Number: 2020-002037-15; Registration date: 26 May 2020.

**Full protocol:**

The full protocol is attached as an additional file, accessible from the Trials website (Additional file 1). In the interest in expediting dissemination of this material, the familiar formatting has been eliminated; this letter serves as a summary of the key elements of the full protocol.

The study protocol has been reported in accordance with the Standard Protocol Items: Recommendations for Clinical Interventional Trials (SPIRIT) guidelines (Additional file 2).

## Supplementary information

**Additional file 1.** Full Study Protocol.

**Additional file 2.** SPIRIT 2013 Checklist: Recommended items to address in a clinical trial protocol and related documents.

## Data Availability

Chief investigators and the corresponding authors will have access to the final trial dataset. The corresponding authors will evaluate any request for data sharing and will consult with the steering committee after the publication of the main results. Requests can be sent to mariabelen.ruiz@salud.madrid.org.

