# Supplementary material for: Sarilumab versus standard of care for the early treatment of COVID-19 pneumonia in hospitalized patients: SARTRE: a structured summary of a study protocol for a randomised controlled trial
Source: Trials. 2020 Sep 16;21:794. doi: 10.1186/s13063-020-04633-3 (PMC7492787; doi:10.1186/s13063-020-04633-3)
Supplement: Supplementary file 1 — Additional file 1. Full Study Protocol. [file 13063_2020_4633_MOESM1_ESM.pdf]

CLINICAL TRIAL PROTOCOL

Multicenter, randomized, open-label study to evaluate the efficacy and safety of SOC + Sarilumab versus Standard of Care for the Early Treatment of COVID-19-pneumonia in Hospitalized Patients.

SARTRE

Protocol Code: SARTRE

Protocol Date: May 05th 2020
Version: 2.0
EudraCT Number: 2020-002037-15

Clinical Trial National Co-coordinators
Dr. Ana Fernández Cruz
Internal Medicine Department. Infectious Diseases Unit
Hospital Universitario Puerta de Hierro Majadahonda

Dr. Aranzazu Sancho López
Clinical Pharmacology Department
Hospital Universitario Puerta de Hierro Majadahonda

CONFIDENTIAL

This document contains confidential information belonging to SARTRE Steering Committee. Acceptance of this document constitutes your agreement that the information contained herein will not be disclosed or anyway communicated to any third part.

This protocol is based on Master Protocol published by the WHO R&D Blueprint Clinical Trials Expert Group. Available in https://www.who.int/blueprint/priority-diseases/key-action/multicenter-adaptive-RCT-of-investigational-therapeutics-for-COVID-19.pdf

PROTOCOL APPROVAL SIGNATURES

I have read the protocol and I shall conduct the Study in strict adherence to the Protocol and the Declaration of Helsinki

Sponsor
Name: Cristina Avendaño Solá, MD, PhD
Clinical Pharmacology Department
Hospital Universitario Puerta de Hierro Majadahonda, Madrid

Date: 05/05/2020
Signature:

Clinical Trial National Co- coordinators:

Name: Ana Fernández Cruz, MD, PhD
Internal Medicine Department
Hospital Universitario Puerta de Hierro Majadahonda, Madrid

Date: 05/05/2020
Signature:

Name: Aránzazu Sancho López, MD, PhD
Clinical Pharmacology Department
Hospital Universitario Puerta de Hierro Majadahonda, Madrid

Date: 05/05/2020
Signature:

Clinical Site Principal Investigator:
Name:
Clinical site:

Date:
Signature:

CONFIDENTIAL

KEY TRIAL CONTACTS

Sponsor	Cristina Avendaño Solà, MD, PhD<br>Servicio de Farmacología Clínica<br>Hospital Univ. Puerta de Hierro Majadahonda<br>C/ Manuel de Falla 1. 28222 Majadahonda, Madrid<br>Phone: 91 191 64 79<br>E-mail: cavendano@salud.madrid.org
Steering Committee	Aránzazu Sancho López, MD, PhD<br>Ana Fernández Cruz, MD, PhD<br>Belén Ruiz-Antorán, MD, PhD<br>Jose Campos Esteban, MD<br>Cristina Avendaño Solá, MD, PhD (*Sponsor*)
Clinical Trial National Co-Coordinators	Aránzazu Sancho López, MD, PhD<br>Hospital Universitario Puerta de Hierro Majadahonda<br>C/ Manuel de Falla 1. 28222 Majadahonda, Madrid<br>Phone: 91 191 70 70<br>E-mail: aranzazu.sancho@salud.madrid.org
	Ana Fernández Cruz, MD, PhD<br>Unidad de Infecciosas, Servicio de Medicina Interna. Hospital Univ. Puerta de Hierro Majadahonda<br>C/ Manuel de Falla 1. 28222 Majadahonda, Madrid<br>Phone: 91 191 60 00<br>E-mail: anafcruz999@gmail.com
Pharmacovigilance	Gustavo Adolfo Centeno Soto, MD, PhD<br>IIS Puerta de Hierro - Segovia de Arana<br>C/ Joaquín Rodrigo 2. 28222. Majadahonda. Madrid<br>Phone: 91 191 6026<br>E-mail: gustavo.centeno@scren.es
Statistical Analysis	Belén Ruiz-Antorán, MD, PhD<br>Servicio de Farmacología Clínica. Hospital Universitario Puerta de Hierro Majadahonda<br>C/ Manuel de Falla 1. 28222 Majadahonda, Madrid<br>Phone: 91 191 74 79<br>E-mail: mariabelen.ruiz@salud.madrid.org
Project Manager	Isabel Salcedo de Diego, RN, PhD<br>Hospital Universitario Puerta de Hierro Majadahonda<br>C/ Manuel de Falla 1. 28222 Majadahonda, Madrid<br>Phone: 91 191 64 81<br>E-mail: Isabel.salcedo@salud.madrid.org

CONFIDENTIAL

Study monitor	Antonio Caballero Bermejo, MD<br>Hospital Universitario Puerta de Hierro Majadahonda<br>C/ Manuel de Falla 1. 28222 Majadahonda, Madrid<br>Phone: 91 191 6480<br>Email: afcaballerobermejo@gmail.com

CONFIDENTIAL

INDEX

1. TRIAL SYNOPSIS 9
2. GENERAL INFORMATION 15
Trial Identification 15
Clinical Trial National Co-Coordinator 15
Project Manager 16
Pharmacovigilance 16
Statistical Analysis 16
Study monitor 16
Investigator Team at Hospital Puerta de Hierro 16
Principal Investigator 16
Centers of the Trial 17
Expected duration of the Trial 17
Patient Number 17
3. BACKGROUND AND RATIONALE FOR THE STUDY 17
4. TRIAL OBJECTIVES 19
Primary Objective 19
Secondary Objectives 19
Objectives and endpoints 19
PRIMARY 19
SECONDARY 19
5. DESIGN OF THE STUDY 21
6. SCREENING AND SELECTION OF STUDY SUBJECTS 23
Inclusion Criteria 23
Exclusion Criteria 24
7. TREATMENT PLAN 24
Method of Treatment Assignment 24
Study drug preparation 26
Potential Risks 26
Administration 26
Handling and Storage 27

CONFIDENTIAL

Dispensing **27**

Accountability **27**

Study Intervention Compliance **28**

Disposal and Destruction **28**

Permitted Concomitant Therapy **28**

Prohibited Concomitant Therapies **28**

Withdrawal from randomized treatment or from the study **28**

Withdrawal criteria **29**

8. STUDY PROCEDURES AND ENDPOINTS ***29***

Study Procedures **29**

Informed Consent 29

Screening and Inclusion (Visit -1) 29

Randomisation and Treatment 31

Efficacy assessments: follow up visits 31

Follow-up period 32

Study outcomes **37**

Primary Endpoint 37

Secondary endpoints 37

Calendar of Evaluation of Trial Variables **37**

9. PHARMACOVIGILANCE/ADVERSE EVENTS/SAFETY ***38***

Definitions **38**

Registry of AE **39**

SAE that do not require expedited notification **39**

Procedure of notification **39**

Reporting of SAEs 39

Notification of Laboratory Abnormalities 40

Clinically Significant (CS) Laboratory Abnormalities 40

Serious Laboratory Abnormalities 40

Urgent or expedited notification of SUSAR to health authorities **40**

Urgent notification of other relevant safety information **40**

Annual safety report **41**

10. STATISTICAL ANALYSIS ***41***

Sample size estimation **41**

Data Review (DR) **41**

Analysis populations **42**

Randomisation Procedure **42**

Statistical analysis **42**

Criteria for ending the trial **43**

Procedures to communicate deviations from planned statistical analysis **43**

CONFIDENTIAL

Subject included in each analysis 43
11. ETHICAL ASPECTS *44*
Good Clinical Practice 44
Ethical considerations 44
Information to the subjects and informed consent 44
Confidentiality of the patients 45
Financial responsibility 45
Insurance 45
Notification to health authorities 45
12. PRACTICAL CONSIDERATIONS *46*
Direct access to data/ source documents 46
Responsibilities of all participants. 46
Audit 47
Use and publication or study results 47
13. FILE KEEPING *47*
Case Report Form (CRF) 47
Documents in custody of the investigator: 47
Trial Master File 47
Monitoring 47
Final Report 47
14. LITERATURE REFERENCES *49*
APPENDIX 1: RESEARCH TEAM *50*
Appendix 2: INFORMED CONSENT/ INFORMATION SHEET *51*
APPENDIX 3: INSURANCE CERTIFICATE *52*
APPENDIX 4: NCI-CTC AE-CRITERIA *53*
APPENDIX 5: SmPC Kevzara *54*
APPENDIX 6: Brescia-COVID Scale *55*
APPENDIX 7: List of Participating Centers *56*

CONFIDENTIAL

ABBREVIATIONS LIST

Abbreviation	Definition
AE	Adverse Event
AEMPS	Agencia Española de Medicamentos y Productos Sanitarios (Spanish Agency of Medicines and MedicalDevices)
ANC	Absolute neutrophil count
AR	Adverse Reaction
ARDS	Acute Respiratory Distress Syndrome
BUN	blood urea nitrogen
CEIm	Comité de ética de la investigación con medicamentos (Research Ethics Committee for medicinal products)
COVID-19	Coronavirus disease 19
CRF	Case report form
CRP	C-reactive protein
CRS	Cytokine Release Syndrome
ICU	intensive care unit
IgG	Human immunoglobulin
IL	Interleukine
RA	Rheumatoid arthritis
RT-PCR	Reverse transcription polymerase chain reaction
SAE	Serious Adverse Event
SRLmb	Sarilumab
SOFA	Sequential Organ Failure Assessment
SOC	Standard of Care
SUSAR	Suspected Unexpected Serious Adverse Response

CONFIDENTIAL

1. TRIAL SYNOPSIS

**Sponsor**	Cristina Avendaño-Solá, MD, PhD. Clinical Pharmacology Department. Hospital Universitario Puerta de Hierro Majadahonda, Madrid
**Clinical Trial National Co-Coordinators**	-Ana Fernández Cruz, MD, PhD. Internal Medicine Department. Hospital Universitario Puerta de Hierro Majadahonda, Madrid<br>-Aránzazu Sancho López, MD, PhD. Clinical Pharmacology Department. Hospital Universitario Puerta de Hierro Majadahonda, Madrid
**Investigational product**	SRLmb is a recombinant human immunoglobulin (IgG) 1 monoclonal antibody that binds specifically to both soluble and membrane-bound interleukin-6 (IL-6) receptors (sIL-6Rα and mIL-6Rα) and inhibits IL-6-mediated signaling. It is approved for the treatment of moderate to severe rheumatoid arthritis. The recommended dose of Sarilumab is 200 mg once every 2 weeks administered as a subcutaneous injection.
**Title of study:**	A multicenter, randomized, open-label study to evaluate the efficacy and safety of sarilumab + Standard of Care versus Standard of Care for the Early Treatment of COVID-19-pneumonia in Hospitalized Patients.
**Rationale for the study**	In some patients, acute, life-threatening respiratory injury produced by viruses such as SARS-CoV and other viral pneumonia are associated with an over-exuberant cytokine release. Elevated levels of blood IL-6 had been identified as a one of the risk factors associated with severe COVID-19 disease.<br>Preliminary experiences with tocilizumab, an anti-IL6 inhibitor, show promising results with improvements seen in clinical symptoms, respiratory function, and inflammatory laboratory parameters.<br>Based on this and on current knowledge on the role of the anti-IL6 inhibitor tocilizumab in the treatment of cytokine release syndrome associated to CART therapies, tocilizumab is being extensively used nowadays in clinical practice, and many clinical trials are being conducted around the world, for preventing the fatal consequences of acute respiratory and multi organ failure associated with acute respiratory syndrome due to severe COVID-19 infection.<br>In Spain, anti-IL6 inhibitors are among the therapeutic armamentarium for preventing the fatal consequences of acute

CONFIDENTIAL

respiratory and multi organ failure in around 20% of the COVID-19 infected patients. At present, their use is prioritized to patients with severe interstitial pneumonia (Brescia-COVID Scale-COVID 2-3) with hyperinflammation as determined by the presence of elevated IL6 and/or d-dimer, or progressive d-dimer increase, in patients who otherwise are subsidiary to ICU admission.

Preliminary results of an unpublished ongoing multicenter, national, observational cohorts Study conducted in patients treated with tocilizumab vs matched controls led by our group suggest that IL-6 inhibitors may prevent progression to ICU admission or death in patients with severe COVID-19 interstitial pneumonia.

However, many uncertainties remain on the actual role of anti-IL6 inhibitors in this setting, and whether current use and timing is the right one. Among clinicians there is the hypothesis that the use of anti-IL6 inhibitors at an earlier state during the hyperinflammatory syndrome would be beneficial and may avoid progressing to ARDS.

On the other hand, the standard of care has changed in the prior weeks and nowadays the use of corticosteroids has become part of the SOC in the treatment of COVID-19 pneumonia, and our limited experience suggests that better treatment outcomes can be achieved when combining IL6-inhibitors with corticosteroids. Thus, the question still remains on whether anti-IL6 inhibitors concomitantly with corticosteroids may potentiate the anti-inflammatory response and lead to better disease outcomes.

The aim of the present study is to evaluate if an earlier therapeutic intervention with sarilumab plus SOC may be more effective than current standard of care alone, which according to our local protocol includes weight adjusted corticosteroids doses, in preventing progression to respiratory failure in COVID-19 infected patients with interstitial pneumonia.

This study will also provide supportive evidence to that provided by currently ongoing studies on the efficacy and safety of sarilumab in this clinical context.

<u>References</u>

1. Geng Li1, Coronavirus infections and immune responses. J Med Virol. 2020;92:424–432.
2. Hasan K. Siddiqi MD, MSCR , Mandeep R. Mehra MD, MSc , COVID-19 Illness in Native and Immunosuppressed States: A Clinical-Therapeutic Staging Proposal, Journal of Heart and Lung Transplantation (2020), doi: https://doi.org/10.1016/j.healun.2020.03.012
3. Xiaoling Xu et al. Effective Treatment of Severe COVID-19

CONFIDENTIAL

	Patients with Tocilizumab. Disponible en: https://www.ser.es/wp-content/uploads/2020/03/TCZ-and-COVID-19.pdf)<br>4. Tratamientos disponibles para el manejo de la infección respiratoria por SARS-CoV-2. AEMPS link: https://www.aemps.gob.es/la-aemps/ultima-informacion-de-la-aemps-acerca-del-covid%e2%80%9119/tratamientos-disponibles-para-el-manejo-de-la-infeccion-respiratoria-por-sars-cov-2/<br>5. Acute Respiratory Distress Syndrome Advances in Diagnosis and Treatment. JAMA. 2018; 319(7):698-710. doi:10.1001/jama.2017.21907
**Phase of development:**	Phase II study
**Primary objective:**	To evaluate the efficacy of an early intervention with sarilumab in the prevention of progression to severe respiratory failure/ICU admission or death in SARS-CoV-2 infected patients with pneumonia and regular oxygen supplement requirements - (Brescia-COVID Score= 1).
**Secondary objectives:**	**Secondary objectives:**<br>1. To assess differences between both strategies in the following:<br>• Time to respiratory failure<br>• Time to reduction of supplemental oxygen requirements<br>• Time to non-invasive or invasive ventilation.<br>• The duration of hospitalization<br>• Mortality rate and survival.<br>• Rate of ICU admission<br>2. To evaluate the efficacy of sarilumab in COVID-19 patients with pneumonia Brescia-COVID- >2 (open-label follow up phase).<br>3. To evaluate safety of sarilumab treatment in treating patients with COVID-19 pneumonia.<br>4. To evaluate differences in the effect of sarilumab on the serum levels of inflammatory cytokines.<br>5. To evaluate differences in the proportion of patients showing more than >1 organ failure, e.g. cardiovascular, liver and kidney failure<br>6. To identify prognosis factors of sarilumab efficacy related to laboratory parameters or disease features.
**Number of patients planned**	To obtain a power of 80.00% to detect differences in the contrast of the null hypothesis H_0: p1 = p2 by means of a bilateral Chi square test for two independent samples, taking into account that the level of significance is 5.00%, and assuming that the

CONFIDENTIAL

	proportion in the Reference group is 25.00%, the proportion in the Experimental group is 10.00%, and a randomization ratio 1.1, it will be necessary to include 100 patients in the Reference group and 100 patients in the Experimental group, with a total of 200 patients in the study.
**Eligibility criteria**	**Inclusion Criteria**<br>1. Patients willing to provide informed consent to participate in this study. Witnessed oral consent will be accepted in order to avoid paper handling. Written consent by patient or representatives will be obtained whenever possible.<br>2. The patient is at least 18 years of age.<br>3. The patient is positive for novel coronavirus by real-time RT-PCR<br>4. The patient is hospitalized for COVID-19 without either mechanical ventilation (invasive or non-invasive) or oxygen mask with reservoir bag and at least one of the following:<br>-Radiographic evidence of pulmonary infiltrates by imaging (chest x-ray, CT scan, etc.),<br>OR<br>-Clinical assessment (evidence of rales/crackles on exam) AND SpO2 ≤ 94% on room air that requires supplemental oxygen.<br>5. More than 7 days between the onset of symptoms (fever, dysnea, and/or cough) and treatment administration day. In the absence of fever, cough, or dyspnea, other symptoms like asthenia, headache, or gastrointestinal symptoms may be considered<br>6. The patients presents progressive elevation of inflammatory parameters suggestive of a hyperinflammatory syndrome:<br>Presence of elevated IL-6 (>40pg/ml)<br>OR<br>Elevated d-dimer (>1.0 mcg/ml), or alternatively, progressive worsening in at least two of these inflammatory parameters in the prior 48h: CRP, LDH, serum ferritin, lymphopenia, or d-dimer.<br>**Exclusion Criteria**<br>1. Requiring mechanical ventilation (invasive or non-invasive) or oxygen mask with reservoir bag at screening.<br>2. Participation in any other clinical trial of an experimental treatment for COVID-19.<br>3. In the opinion of the clinical team, progression to death is imminent and inevitable within the next 24 hours, irrespective of the provision of treatments.

CONFIDENTIAL

	4. Any incompatibility or allergy to the administration of sarilumab or corticosteroids.
**Study drug dose**	Sarilumab will be administered intravenously following this posology:<br><br>-Patients <75kg body weight: single 200mg SARILUMAB dose (1 single-dose pre-filled syringe containing 200 mg sarilumab in 1.14 ml solution (175 mg/ml) added to 100 mL 0.9% sodium chloride for a 1-hour intravenous infusion).<br><br>-Patients ≥75kg body weight: single 400mg SARILUMAB dose (2 single-dose pre-filled syringes, each containing 200 mg sarilumab in 1.14 ml solution (175 mg/ml) added to 100 mL 0.9% sodium chloride for a 1-hour intravenous infusion).<br><br>In addition, all study participants will receive treatment with corticosteroids intravenously following this posology: Methylprednisolone 1mg/kg/day (or corticosteroid dose equivalents) for at least 3 days<br><br>Sarilumab plus corticosteroids or corticosteroids alone will be given in add-on to SOC medication. SOC medication will be based on local clinical practice recommendations.
**Duration of study**	The observation period will be from randomization until 28 days after randomisation, hospital discharge, ICU admission or death, whichever occurs first.
**General design and methodology**	A multi-center, randomized, clinical trial with two arms to study the efficacy and safety of early treatment with sarilumab plus SOC compared to standard of care (SOC) in patients with COVID-19 pneumonia (BCRSS score 1).<br><br>Experimental arm: Sarilumab + methylprednisolone (or equivalent doses) plus SOC for COVID-19<br><br>Control arm: Methylprednisolone (or equivalent doses) plus SOC for COVID-19.<br><br>Randomization will be 1:1 to any of the treatment groups and will be stratified per center.<br><br>Methylprednisolone 1mg/kg/day (or corticosteroid dose equivalents) for at least 3 days.<br><br>SOC includes any drugs that are being used in clinical practice (e.g. lopinavir/ritonavir; hydroxy/chloroquine, and other than those used as part of another clinical trial) according to each site local protocol.<br><br>Patients will be followed up to four weeks after randomization.<br><br>Patients randomized to the control arm (CS + SOC group without sarilumab) progressing to severe respiratory failure fulfilling criteria for treatment with anti-IL6 inhibitors according to clinical practice guidelines, as defined by the presence of Brescia-COVID

CONFIDENTIAL

	Scale 2-3 plus inflammatory markers, will be offered the option to be rescued with sarilumab at the same doses and be included in an open-label follow-up phase. This group of patients will be followed up for two additional weeks following sarilumab administration.<br>The results of the study will also be compared with an external cohort of matched patients that did not receive IL-6 inhibitors. This cohort of patients is already being collected as part of an academic multicenter, national, prospective observational cohorts Study being conducted by the same investigation team. This will provide further contextualization to our results and facilitate interpretation.
**Primary measure and endpoint:**	The primary endpoint is the proportion of patients progressing to severe respiratory failure (Brescia-COVID Scale ≥2), ICU admission, or death.
**Secondary measures and endpoints**	Time to progression to severe respiratory failure (Brescia-COVID ≥2)<br>Time to reduction of supplemental oxygen requirements<br>Time to non-invasive or invasive mechanical ventilation.<br>Rate of hospital discharge and duration of hospitalization<br>Mortality rate and survival time<br>Rate of ICU admission<br>Proportion of patients with at least 1 point progression in the 7-point ordinal scale<br>*Ordinal scale:*<br>*1) Not hospitalized, no limitations on activities.*<br>*2) Not hospitalized, limitation on activities.*<br>*3) Hospitalized, not requiring supplemental oxygen.*<br>*4) Hospitalized, requiring supplemental oxygen.*<br>*5) Hospitalized, on non-invasive ventilation or high flow oxygen devices or oxygen mask with reservoir bag.*<br>*6) Hospitalized, on invasive mechanical ventilation or ECMO.*<br>*7) Death.*<br><br>Changes from baseline to day 15th in serum levels of inflammatory cytokines.<br>Proportion of patients showing more than >1 organ failure, e.g. cardiovascular, liver and kidney failure<br>Safety and tolerability – Adverse events

CONFIDENTIAL

2. GENERAL INFORMATION

Trial Identification

Protocol Code: SARTRE

EUDRACT: 2020-002037-15

Version and Date: v 2.0 of May 5th 2020

Title of the trial: Multicenter, randomized, open-label study to evaluate the efficacy and safety of SOC + sarilumab versus Standard of Care for the Early Treatment of COVID-19-pneumonia in Hospitalized Patients.

Steering Committee

Ana Fernández Cruz, MD, PhD

Aránzazu Sancho López, MD, PhD

Elena Muñez Rubio, MD, PhD

Antonio Ramos Martínez, MD, PhD

Belén Ruiz-Antorán, MD, PhD

Jose Campos Esteban, MD

Cristina Avendaño Solá, MD, PhD (Sponsor)

Clinical Trial National Co-Coordinators

-Ana Fernández Cruz, MD, PhD

Unidad de Infecciosas, Servicio de Medicina Interna. Hospital Univ. Puerta de Hierro Majadahonda

C/ Manuel de Falla 1. 28222 Majadahonda, Madrid

Phone: 91 191 60 00

E-mail: anafcruz999@gmail.com

-Aránzazu Sancho López, MD, PhD

Hospital Universitario Puerta de Hierro Majadahonda

C/ Manuel de Falla 1. 28222 Majadahonda, Madrid

Phone: 91 191 70 70

E-mail: aranzazu.sancho@salud.madrid.org

CONFIDENTIAL

Project Manager

Isabel Salcedo de Diego, RN, PhD

Hospital Universitario Puerta de Hierro Majadahonda

C/ Manuel de Falla 1. 28222 Majadahonda, Madrid

Phone: 91 191 64 81

E-mail: Isabel.salcedo@salud.madrid.org

Pharmacovigilance

Gustavo Adolfo Centeno Soto, MD, PhD

IIS Puerta de Hierro - Segovia de Arana

C/ Joaquín Rodrigo 2. 28222. Majadahonda. Madrid

Phone: 91 191 6026

E-mail: gustavo.centeno@scren.es

Statistical Analysis

Belén Ruiz-Antorán, MD, PhD

Servicio de Farmacología Clínica. Hospital Universitario Puerta de Hierro Majadahonda

C/ Manuel de Falla 1. 28222 Majadahonda, Madrid

Phone: 91 191 74 79

E-mail: mariabelen.ruiz@salud.madrid.org

Study monitor

Antonio Caballero Bermejo, MD

Hospital Universitario Puerta de Hierro Majadahonda

C/ Manuel de Falla 1. 28222 Majadahonda, Madrid

Phone: 91 191 6480

Email: afcaballerobermejo@gmail.com

Investigator Team at Hospital Puerta de Hierro

Principal Investigator

Ana Fernández Cruz, MD, PhD. Internal Medicine

CONFIDENTIAL

Co-investigators

- *Internal Medicine Department*

Antonio Ramos, MD, PhD

Elena Muñez MD, PhD

Alejandro Callejas MD, PhD

Pablo Tutor MD, PhD

Susana Mellor MD, PhD

Víctor Moreno-Torres MD

Isolina Baños MD, PhD

- *Rheumatology Department*

Jesús Sanz Sanz, MD

- *Clinical Pharmacology Department*

Elena Diago Sempere MD

Isabel Salcedo de Diego, RN, PhD

Centers of the Trial

This will be a multicenter national study, with Hospital Universitario Puerta de Hierro Majadahonda acting as Study Site Coordinator. The list of participating centers can be found attached as Appendix 7.

Expected duration of the Trial

Expected recruitment will take 2 months since first site initial visit, which will follow the approval of the AEMPS.

Each patient will participate in the trial for a maximum of 29 days, including a screening phase of 1 day, treatment duration of 1-2 day, initial follow-up and a follow-up visit at 28 days from randomisation.

Patient Number

Two hundred patients

3. BACKGROUND AND RATIONALE FOR THE STUDY

Drug Information

Kevzara® (sarilumab) is a fully human IgG1 monoclonal antibody that binds specifically to both soluble and membrane-bound IL-6 receptors (sIL-6Rα and mIL-6Rα) and has been shown to inhibit IL-6-

CONFIDENTIAL

mediated signaling through these receptors. IL-6 is a pleiotropic pro-inflammatory cytokine produced by a variety of cell types including T- and B-cells, lymphocytes, monocytes and fibroblasts. IL-6 has been shown to be involved in diverse physiological processes such as migration and activation of T-cells, induction of immunoglobulin secretion, initiation of hepatic acute phase protein synthesis, and stimulation of hematopoietic precursor cell proliferation and differentiation.

Kevzara® (sarilumab) is authorized in the European Union for the treatment of adult patients with moderately to severely active rheumatoid arthritis (RA) who have had an inadequate response or intolerance to one or more biologic or non-biologic disease-modifying anti-rheumatic drugs (DMARDs). The recommended dose is 200 mg once every 2 weeks given as a subcutaneous injection.

Study rationale

In some patients, acute, life-threatening respiratory injury produced by viruses such as SARS-CoV and other viral pneumonia are associated with an over-exuberant cytokine release. Elevated levels of blood IL-6 had been identified as a one of the risk factors associated with severe COVID-19 disease. Preliminary experiences with tocilizumab, an anti-IL6 inhibitor, show promising results with improvements seen in clinical symptoms, respiratory function, and inflammatory laboratory parameters.

Based on this and on current knowledge on the role of the anti-IL6 inhibitor tocilizumab in the treatment of cytokine release syndrome associated to CART therapies, tocilizumab is being extensively used nowadays in clinical practice, and many clinical trials are being conducted around the word, for preventing the fatal consequences of acute respiratory and multi organ failure associated with acute respiratory syndrome due to severe COVID-19 infection.

In Spain, anti-IL6 inhibitors are among the therapeutic armamentarium for preventing the fatal consequences of acute respiratory and multi organ failure in around 20% of the COVID-19 infected patients. At present, their use is prioritized to patients with severe interstitial pneumonia (COVID2-3) with hyperinflammation as determined by the presence of elevated IL6 and/or d-dimer, or progressive d-dimer increase, in patients who otherwise are subsidiary to ICU admission.

Preliminary results of an unpublished ongoing multicenter, national, observational cohorts Study conducted in patients treated with tocilizumab vs matched controls led by the same local investigation team suggest that IL-6 inhibitors may prevent progression to ICU admission or death in patients with severe COVID-19 interstitial pneumonia and that there is a window of opportunity for this drug, yet to be determined.

However, many uncertainties remain on the actual role of anti-IL6 inhibitors in this setting, and whether current use and timing is the right one. Among clinicians there is the hypothesis that the use of anti-IL6 inhibitors at an earlier state during the hyperinflammatory syndrome than the current one would be beneficial and may avoid progressing to ARDS.

On the other hand, the standard of care has changed in the prior weeks and nowadays the use of corticosteroids has become part of the SOC in the treatment of COVID-19 pneumonia, and our limited experience suggests that better treatment outcomes can be achieved when combining IL6-inhibitors with corticosteroids. Thus, the question still remains on whether anti-IL6 inhibitors concomitantly with corticosteroids may potentiate the anti-inflammatory response and lead to better treatment outcomes.

Hypothesis

CONFIDENTIAL

The hypothesis of this trial is that in patients with pneumonia and respiratory failure caused in the setting of COVID-19, an early blockade of IL-6 with sarilumab plus weight adjusted corticosteroid doses plus SOC, will attenuate the cytokine storm and halt the progression to acute respiratory distress syndrome (ARDS) and the need for mechanical ventilation, ICU admission, or death.

4. TRIAL OBJECTIVES

Primary Objective

The overall objective of the study is to evaluate the efficacy of an early intervention with sarilumab in the prevention of progression to severe respiratory failure/ICU admission or death in COVID-19 infected patients with pneumonia and regular oxygen supplement requirements (Brescia-COVID- 1).

Secondary Objectives

1. To assess differences between both strategies in the following:
   - Time to respiratory failure
   - Time to reduction of supplemental oxygen requirements
   - Time to mechanical non-invasive or invasive ventilation.
   - The duration of hospitalization
   - Mortality rate and survival.
   - Rate of ICU admission
   - To evaluate the efficacy of sarilumab in COVID-19 infected patients with pneumonia Brescia-COVID ≥2 (open-label follow up phase).
2. To evaluate safety of sarilumab treatment in treating patients with COVID-19 pneumonia.
3. To evaluate differences in the effect of sarilumab on the serum levels of inflammatory cytokines in patients with COVID-19.
4. To evaluate differences in the proportion of patients showing more than >1 organ failure, e.g. cardiovascular, liver and kidney failure
5. To identify prognosis factors of sarilumab efficacy related to laboratory parameters or disease features

Objectives and endpoints

Table 1 - Objectives and endpoints

OBJECTIVES	ENDPOINTS AND OUTCOME MEASURES
PRIMARY	
**Evaluate the efficacy of an early intervention with sarilumab in COVID-19 infected patients with pneumonia and regular oxygen supplement requirements (Brescia-COVID 1)**	The primary endpoint is the proportion of patients progressing to severe respiratory failure (Brescia-COVID ≥2), ICU admission or death from randomization up to day 15th of follow-up.
SECONDARY	

CONFIDENTIAL

**Evaluate effects of efficacy of an early intervention with sarilumab on secondary efficacy endpoints as compared to standard of care as assessed by:**	
• Clinical Severity: progression to severe respiratory failure/ICU admission or death	Time to respiratory failure<br>Rate of ICU admission<br>Rate of mortality
• Oxygenation:<br>o Oxygenation free days in the first 15 days<br>o Incidence and duration of new oxygen use during the study<br>o Mechanical Ventilation<br>• Ventilator free days in the 15 days.<br>• Incidence and duration of new mechanical ventilation use during the study	Time to reduction of supplemental oxygen requirements<br>Time to non-invasive or invasive ventilation.<br><br>Proportion of patients with at least 1point progression in the 7-point ordinal scale from baseline up to day 15<br>*Ordinal scale:*<br>*1) Not hospitalized, no limitations on activities.*<br>*2) Not hospitalized, limitation on activities.*<br>*3) Hospitalized, not requiring supplemental oxygen.*<br>*4) Hospitalized, requiring supplemental oxygen.*<br>*5) Hospitalized, on non-invasive ventilation or high flow oxygen devices or oxygen mask with reservoir bag.*<br>*6) Hospitalized, on invasive mechanical ventilation or ECMO.*<br>*7) Death.*
• Hospitalization<br>• Mortality<br>o 15-day mortality	Duration of hospitalization.<br><br>Date and cause of death (if applicable).
**Evaluate the efficacy of sarilumab in in COVID-19 infected patients with pneumonia Brescia-COVID- ≥2 (open-label follow up phase).**	Rate of ICU admission<br>Rate of mortality
**To evaluate safety of sarilumab as compared to SOC**<br>Safety through day 29<br>• Cumulative incidence of serious adverse events (SAEs)<br>• Cumulative incidence of Grade 3 and 4 adverse events (AEs).	Serious adverse events (SAEs).<br>Grade 3 or 4 Adverse events (AEs).

CONFIDENTIAL

**To evaluate differences in the effect of sarilumab on the serum levels of inflammatory cytokines in patients infected with COVID-19**.	Changes in serum level of CRP, IL-6, lymphocyte count, neutrophils count, d-dimer, LDH, ferritin, coagulation tests
**To evaluate differences in the proportion of patients showing more than >1 organ failure, e.g. cardiovascular, liver and kidney failure**	Patients showing more than >1 organ failure through day 15
**To identify prognosis factors of sarilumab efficacy related to laboratory parameters or disease features**	Focused medical history, demographic characteristics, COVID-19 history, concomitant medication, and laboratory disease parameters.

5. DESIGN OF THE STUDY

This is an exploratory phase II, multicenter, open-label, randomized clinical trial to evaluate the efficacy and safety of an early intervention with sarilumab + methylprednisolone (or equivalent CS dose) plus SOC vs methylprednisolone (or equivalent CS dose) plus SOC alone in patients with COVID-19 pneumonia aimed to prevent progression to severe respiratory failure, ICU admission, or death.

Experimental arm: Sarilumab + methylprednisolone (or equivalent CS doses) plus SOC for COVID-19

Control arm: methylprednisolone plus SOC for COVID-19.

Sarilumab will be administered intravenously following this posology:

-Patients <75kg body weight: single 200mg SARILUMAB dose (1 single-dose pre-filled syringe containing 200 mg sarilumab in 1.14 ml solution (175 mg/ml) added to 100 mL 0.9% sodium chloride for a 1-hour intravenous infusion).

-Patients ≥75kg body weight: single 400mg SARILUMAB dose (2 single-dose pre-filled syringes, each containing 200 mg sarilumab in 1.14 ml solution (175 mg/ml) added to 100 mL 0.9% sodium chloride for a 1-hour intravenous infusion).

Corticosteroids will be given to all patients at a 1mg/kg/d of methylprednisolone (or equivalent CS dose) for at least 3 days.

SOC includes~~ing~~ weight adjusted corticosteroid doses plus any drugs that are being used in clinical practice (e.g. lopinavir/ritonavir; hydroxy/chloroquine, and other than those used as part of another clinical trial

Randomization will be 1:1 to any of the treatment groups and will be stratified per center.

For patients on will be rescued with sarilumab at the same doses and be included in a 15-day open-label follow up phase or until hospital discharge, ICU admission or death.

Clinical follow-up visits will be performed at 3, 5, and 15 days after treatment randomization.

Patients in any treatment group progressing to severe respiratory failure fulfilling criteria for treatment with anti-IL6 inhibitors according to clinical practice guidelines, as defined by the presence of Brescia-COVID 2-3 plus inflammatory markers, will be considered treatment failures for the sake

CONFIDENTIAL

of the primary analysis and be rescued according to local treatment protocols, which may include tocilizumab. Patients in the control group (SOC group without sarilumab) progressing to Brescia-COVID 2-3 plus inflammatory markers, will be given the option to be rescued with sarilumab at the same doses and, in that case, be included in an open-label phase and be followed up for two additional weeks (with visits at 3, 7 and 15 days after sarilumab rescue administration).

A final follow-up visit will be conducted for all patients at D29 from randomization, regardless of initial treatment assignment.

Figure 1 – Graphical study design

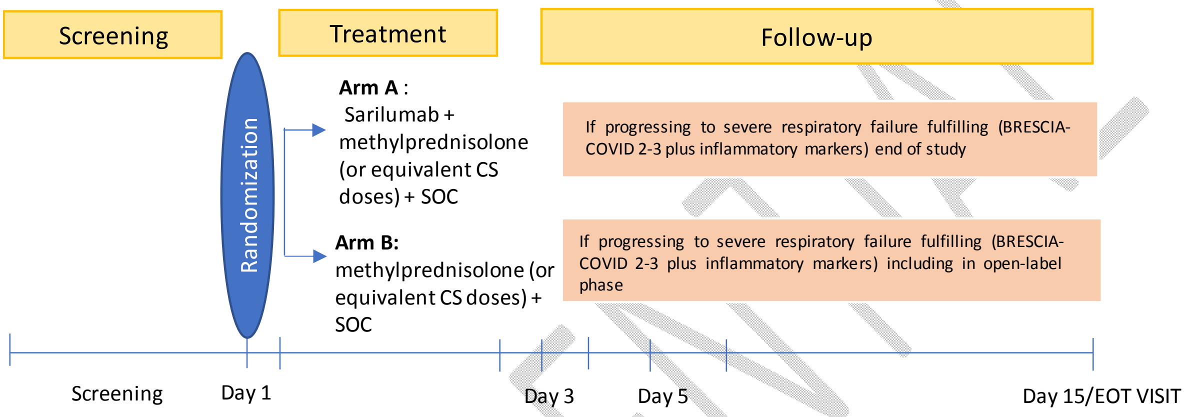

CONFIDENTIAL

Figure 2 – Graphical study design OPEN-LABEL PHASE

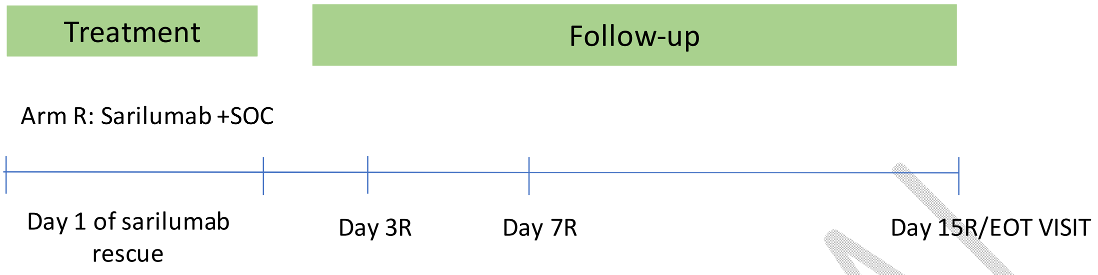

6. SCREENING AND SELECTION OF STUDY SUBJECTS

Inclusion Criteria

1. Informed consent prior to performing any study procedure. Witnessed oral consent will be accepted in order to avoid paper handling. Written consent by patient or representatives will be obtained whenever possible.
2. Male or female adult patients of at least 18 years of age.
3. The patient is positive for SARS-CoV-2 by real-time PCR or other validated tests
4. The patient is hospitalized for COVID-19 without either mechanical ventilation (invasive or non-invasive) or oxygen mask with reservoir bag and at least one of the following:

   -Radiographic evidence of pulmonary infiltrates by imaging (chest x-ray, CT scan, etc.),

   OR

   -Clinical assessment (evidence of rales/crackles on exam) AND SpO2 ≤ 94% on room air that requires supplemental oxygen.
5. More than 7 days between the onset of symptoms (fever, cough, and/or dyspnea) and treatment administration day. In the absence of fever, cough, or dyspnea, other symptoms like astenia, headache, or gastrointestinal symptoms may be considered.
6. The patient present progressive elevation of inflammatory parameters suggestive of a hyperinflammatory syndrome :

   - Presence of elevated IL-6 (>40 pg/mL)

   or

   -Elevated d-dimer (>1.0 mcg/ml), or alternatively, progressive worsening in at least two of these inflammatory parameters in the prior 24-48h: CRP, LDH, serum ferritin, lymphopenia, or d-dimer.
7. Patient must be, in the investigator opinion, able to comply with all the protocol procedures.
8. Negative pregnancy test in case of fertile women

CONFIDENTIAL

Exclusion Criteria

1. Patients with high oxygen requirements including face mask with reservoir, non-invasive mechanical ventilation or high flow nasal cannula, or mechanical ventilation.
2. Patients admitted at the ICU
3. Participation in any other clinical trial of an experimental treatment for COVID-19.
4. In the opinion of the clinical team, progression to death is imminent and inevitable within the next 24 hours, irrespective of the provision of treatments.
5. Any hypersensitivity or allergy to the administration of sarilumab or corticosteroids.
6. Current treatment with immunosuppressive or any immunomodulatory medication within 8 weeks of baseline.
7. Patients who have received immunosuppressive antibody therapy within the past 5 months, including intravenous immunoglobulin
8. AST/ALT values > 10 x ULN
9. Neutropenia (< 0.5 x 109/L).
10. Severe thrombocytopenia (< 50 x 109/L).
11. Sepsis caused by an alternative pathogen.
12. Diverticulitis with risk of perforation.
13. Ongoing infectious dermatitis.
14. Patients with another active infection, including localized infections.
15. Pregnant or breast-feeding females will be excluded

7. TREATMENT PLAN

Method of Treatment Assignment

This is a multicenter, randomized, open label clinical trial evaluating the safety and efficacy of sarilumab (Kevzara®) for the early treatment of the hyperinflammatory syndrome caused by the infection with SARS CoV 2.

Eligible patients shall be enrolled in the study under the supervision of the investigator or designated sub-investigators.

This open-label trial will use blind randomization of patients in a 1:1 ratio to sarilumab (plus corticosteroids) or SOC (plus corticosteroids) through a centralized system embedded in the eCRF (Oracle Clinical). Randomization will be stratified by site.

Dosing and Administration

<u>Experimental group</u>: Patients in the treatment arm will receive sarilumab plus corticosteroids on top of SOC medication. Sarilumab will be administered intravenously as a single dose following this posology:

CONFIDENTIAL

-Patients <75kg body weight: single 200mg SARILUMAB dose (1 single-dose pre-filled syringe containing 200 mg sarilumab in 1.14 ml solution (175 mg/ml) added to 100 mL 0.9% sodium chloride for a 1-hour intravenous infusion).

-Patients ≥75kg body weight: single 400mg SARILUMAB dose (2 single-dose pre-filled syringes, each containing 200 mg sarilumab in 1.14 ml solution (175 mg/ml) added to 100 mL 0.9% sodium chloride for a 1-hour intravenous infusion).

- Corticosteroids will be administered intravenously following this posology: Methylprednisolone 1mg/kg/day (or corticosteroid dose equivalents) for at least 3 days

<u>Control group</u>: Patients in the control group will receive corticosteroids on top of SOC medication.

Corticosteroid: will be administered intravenously following this posology: Methylprednisolone 1mg/kg/day (or corticosteroid dose equivalents).

SOC: All patients will continue background SOC according to local clinical practice protocols and be followed up until disease progression, patient withdrawal, ICU admission, hospital discharge, loss of follow up, or death.

Pharmaceutical Information

Storage, distribution, and usage of these materials in accordance with the protocol and any applicable laws and regulations.

Sarilumab (Kevzara®) pharmaceutical information according to authorized SmPC

Dose formulation:

Sarilumab 200mg: one single-dose pre-filled syringe containing 200mg sarilumab in 1.14 ml solution (175 mg/ml) added to 100mL 0.9% sodium chloride for a 1-hour intravenous infusion. Unit dose strength: 200mg

Sarilumab 400mg: two single-dose pre-filled syringe containing 200mg sarilumab in 1.14 ml solution (175 mg/ml) added to 100mL 0.9% sodium chloride for a 1-hour intravenous infusion. Unit dose strength: 400mg

Route of administration: intravenous

Storage: each box will be stored at the site at a temperature of 2ºC to 8ºC

Packaging and labelling: Study intervention will be provided in box by Sanofi. Each box will contain 2 prefilled syringes and will be labelled as required by local regulation.

Dosing instructions:

After removing the syringes from the refrigerator, they should be allowed to reach room temperature (<25°C).

Before starting the dilution procedure, the clinical center (Pharmacist or Nurse) will check that the syringes used for dilution do not contain any particles or discoloration. If any particulate matter, cloudiness or discoloration is detected, the syringe is to be quarantined, prohibited from use and reported to the Sponsor. IMP will not be removed.

CONFIDENTIAL

Sarilumab prefilled syringe (1or 2 syringes for 200 mg or 400 mg dosage, respectively) will be diluted under aseptic conditions in 100 mL 0.9% Sodium Chloride solution bag.The infusion bag will be turned upside down at least three times to ensure a good mixing of the solutions.

The infusion will be administered within 30 minutes from the end of preparation (as no mixture stability is known). The product will be infused using a volumetric pump at the protocol-specific use and reported to the Sponsor. IMP will not be removed from the box until just before dosing in order to protect from light.

Incompatibilities: this medicinal product must not be mixed with other medicinal products.

Manufacturer: Sanofi-Aventis Groupe

Study drug preparation

The local hospital pharmacist or nurse will prepare the treatment dose required for each patient. Individual treatment dose is prepared in the preparation area of the pharmacy, at a workstation free from any items not needed for this operation, and will be performed in aseptic conditions, as per local rules. Safety measures for preparation and handling include protective clothing, gloves, and safety cabinets.

Potential Risks

Serious infections:

Patients should be closely monitored for the development of signs and symptoms of infection during treatment with Kevzara®. As there is a higher incidence of infections in the elderly population in general, caution should be used when treating the elderly.

Laboratory abnormalities:

The activity of sarilumab in reducing inflammation is associated with laboratory changes such as decrease in ANC and elevation in lipids.

Please refer to the SmPC for additional precautionary measures during sarilumab treatment (Appendix 5)

Administration

Premedication is not required prior to the administration of sarilumab unless deemed medically necessary by the investigator.

Any participant experiencing an Infusion-related reaction (IRR) must receive appropriate medical treatment. When the participant's condition is stable, the infusion may be restarted at a slower rate. In general, upon restarting, the infusion rate must be decreased by half at the time the infusion was interrupted.

Packaging and Labeling

All study drugs will be administered as open label. No blinding methods will be used in this trial.

Experimental drugs will be supplied to pharmacies packaged and labeled according to the standard requirements for clinical trials. Sarilumab will be labelled with the study protocol code, batch number, content, expiry date, storage conditions, investigator and sponsor name. The study medication will be labelled in accordance with annex 13 of the European Good Manufacturing

CONFIDENTIAL

Practices.

Each infusion bag will be clearly marked with the following information:

- Investigational site study code / Sponsor study code:
- Product code:
- Route of administration: For IV administration
- Patient number:
- Dose: 200 mg or 400mg unit
- For clinical trial use only

Handling and Storage

All study treatment supplies must be stored in accordance with the manufacturer's instructions and package labeling. Until dispensed to the participants, the study treatment will be stored in a securely locked area, accessible to authorized personnel only. All study interventions must be stored in a secure, environmentally controlled, and monitored (manual or automated) area in accordance with the labeled storage conditions with access limited to the investigator and authorized site staff.

Upon receipt, sarilumab will be placed into a refrigerator and stored between +2°C and +8°C.

The investigator or designee must confirm appropriate temperature conditions have been maintained during transit for all study intervention received and any discrepancies are reported and resolved before use of the study intervention.

If a temperature excursion occurs:

- The affected products will be placed in quarantine immediately with a clear sign of "quarantined" and clearly separated from the rest of the supplies. In addition, the affected products will be stored under proper conditions as per storage requirements.
- The Site Monitor will be informed about the temperature excursion within the same business day.

Dispensing

Only participants enrolled in the study may receive study intervention and only authorized site staff may supply or administer study intervention. The investigator agrees that study drug will be dispensed by the investigator or sub-investigator(s) named on the Investigator Agreement or their qualified designees. The investigator, sub-investigators, or qualified designees also agree that the study drug(s) will be dispensed only to study subjects who have provided written informed consent (or oral consent where appropriate) and have met all entry.

Accountability

The investigator or the institution, is responsible for study intervention accountability, reconciliation, and record maintenance (i.e., receipt, reconciliation, and final disposition records).

An accurate accountability record of products received, dispensed, discarded and returned or

CONFIDENTIAL

destroyed by the pharmacy at the investigator site will be maintained on pharmacy file.

Study Intervention Compliance

Participants will receive study intervention directly from the investigator or designee, under medical supervision. The date and time of each dose administered in the clinic will be recorded in the source documents and reported in the case report form (CRF). The dose of study intervention and study participant identification will be confirmed at the time of dosing by a member of the study site staff other than the person administering the study intervention.

Sarilumab will be intravenously administered to participants at the site.

Disposal and Destruction

Precaution will be taken to avoid direct contact with the study interventions. In the case of unintentional occupational exposure notify the Medical Monitor.

Sarilumab is for single use only. Any waste material should be disposed of in accordance with local requirements. Unused medicinal product can be used as rescue medication of the study participants. Non-used medication will be returned to Sanofi or kept at local pharmacy for regular use upon Sponsor agreement. Medication will not be destroyed.

Permitted Concomitant Therapy

Participants should receive full supportive care during the study, as well as treatment with antibiotics and analgesics, as appropriate. All concomitant therapies will be permitted. No restriction is established regarding concomitant treatment.

Only clinical relevant concomitant treatment related to COVID-19 will be recorded for the purposes of the trial. The list of concomitant medications will be assessed from 8 weeks prior to enrolment to end of study.

Prohibited Concomitant Therapies

The following medications are prohibited while receiving protocol therapy:

- Concomitant administration of tocilizumab.
- Any investigational agents other than sarilumab.
- Concurrent use of live vaccines.
- Other immunomodulators or immunosuppressant medications.

Withdrawal from randomized treatment or from the study

Patients are free to withdraw from participation in the study at any time. Patients are listed as having withdrawn consent only when they no longer wish to participate in the study and no longer authorize the Investigators to make efforts to continue to obtain their outcome data. Every effort should be made to encourage patients to remain in the study for the duration of their planned outcome assessments. In the case of a patients becoming lost to follow-up, attempts to contact the patient be made and documented in the patient’s medical records.

Treatment discontinuation is not foreseen given that sarilumab therapy will be given as a single dose administration during the initial randomized phase. For patients progressing to severe

CONFIDENTIAL

respiratory failure, the study foresees the possibility to administer sarilumab as rescue therapy under certain conditions. Patients may decide not to receive sarilumab in this second phase of the study without any justification, but efforts should be made by investigators to collect reasons behind and to continue to obtain their outcome data. Investigators may choose to discontinue treatment in this second phase due to severe toxicity to sarilumab during prior administration or due to any other medical reason for which treatment with sarilumab is not considered a suitable choice for the patient.

Withdrawal criteria

A patient may be removed from the study treatment for the following reasons; however, whenever possible the patient should be followed regardless of their protocol adherence as per the efficacy and safety evaluations:

- Patient withdraws consent or requests discontinuation from the study for any reason
- Termination of the study
- Lost to follow-up.
- Due to toxicity, depending on the severity and the relationship of the adverse event to the experimental therapy.

Patients who withdraw from this study or are lost to follow-up after signing the informed consent form (ICF) (or giving oral consent) and administration of the study product, will not be replaced. The reason for patient discontinuation from the study will be recorded on the appropriate case report form.

8. STUDY PROCEDURES AND ENDPOINTS

Study Procedures

Informed Consent

Patients with suspicion of COVID-19 pneumonia will be evaluated by the investigators at the participating sites. Oral consent will be accepted in order to avoid paper handling. Investigator must reflect the process of obtaining consent in the patient's clinical documentation. Written consent by patient or representatives will be obtained whenever possible.

Screening and Inclusion (Visit -1)

Patients will be screened in the hospitalization ward by the investigators of the trial. Screening will take place within 1 day of selection, and administration of the first dose of sarilumab will be done within the following 24 hours. Screening will be preceded by a presentation of the complete information about the clinical study to the subject or family member by the investigator followed by signature of the informed consent form or by obtaining oral consent.

After the informed consent, all the following assessments are performed to determine eligibility requirements as specified in the inclusion and exclusion criteria and before the patient receives any study medication:

-Positive SARS-CoV-2 test result. Laboratory-confirmed SARS-CoV-2 infection as determined by PCR in naso/oropharyngeal swabs or any other relevant.

CONFIDENTIAL

-Focused medical history, including the following information:

- Patient demographic characteristics: age, sex, race
- History of relevant chronic medical conditions and concomitant medications
- COVID-19 history: date of confirmation of disease, disease duration, fever, cough, other related symptoms.
- COVID-19 background medication: Corticosteroids, retrovirals, Hidroxychloroquine, etc.

-Focused physical examination: including vital signs, weight, auscultation, signs of respiratory distress, and any other as clinically indicated

-Oxygen supplement requirements: type and FiO2

-Oxygen parameters: SaO2, PaO2 (if available), SaO2 or PaO2/FiO2 Indexes

-Review recent radiographic imaging (x-ray or CT scan), valid up to 96h prior to randomisation.

-Laboratory disease parameters (IL-6, LDH, d-dimer, CRP, BUN, Ferritin, total white blood cell and total lymphocytes count, platelets count, and liver enzyme studies), valid within 24h prior to randomization

-Optional blood samples will be collected at baseline for future pharmacogenomic exploratory research*

-Pregnancy Test (Pregnancy test in women of childbearing potential)

-Brescia-COVID Scale, CURB-65

-Adverse events

The overall eligibility of the subject to participate in the study will be assessed once all screening values are available. The screening process can be suspended prior to complete assessment at any time if exclusions are identified by the study team. Study subjects who qualify will be immediately randomized and treatment should be administered within 24 hours after randomization.

*Optional blood samples for exploratory research. For patients agreeing to participate and signing the specific biobank informed consent, two additional blood samples will be collected at baseline:

- One yellow tube with gel (5 ml), to collect serum after centrifugation.
- One violet EDTA tube (5 mL) to collect plasma and Buffy Coat after centrifugation.

Specific details to collect and store these samples will be provided to all participating centers.

Note: the data of all the procedures carried out as part of the customary healthcare before the informed consent shall be recorded in the study and do not have to be repeated after the consent is given, if they are obtained in a time period of 48h.

CONFIDENTIAL

Randomisation and Treatment

Participants will be randomly assigned to:

-Treatment arm: sarilumab 200mg if <75kg body weight or 400mg if ≥75kg body weight, corticosteriods plus SOC for COVID-19

-Control arm: corticosteroids plus SOC for COVID-19.

Corticosteroids will be administered intravenously following this posology: Methylprednisolone 1mg/kg/day (or equivalent CS doses) for at least 3 days plus SOC according to local clinical practice protocols.

Study participants will be assigned using a central randomization service implemented in the eCRF. Randomization codes will be provided by the CRO and will be charged into the eCRF as to maintain the treatment concealment.

The following assessments will be performed before study drug administration to confirm selection criteria:

-Vital signs including SpO2 and Tª

-Focused physical examination: including vital signs, weight, auscultation, signs of respiratory distress, and any other as clinically indicated

-Oxygen supplement requirements: type and FiO2

-Oxygen parameters: SaO2, PaO2 (if available), SaO2 or PaO2/FiO2

Efficacy assessments: follow up visits

Clinical follow-up visits will be performed at 3, 5, and 15 days after treatment randomisation Patients in SOC group without sarilumab progressing to severe respiratory failure fulfilling criteria for treatment with anti-IL6 inhibitors according to clinical practice guidelines, as defined by the presence of Brescia-COVID 2-3 plus inflammatory markers, will be rescued with sarilumab at the same doses and be included in an open-label phase. This group of patients will be followed up by two additional weeks after receiving sarilumab as recue medication (days 3, 5 and 15 following salirumab administration).

Patients randomly assigned to sarilumab therapy at baseline progressing to Brescia-COVID 2-3 will be rescued according to local clinical practice protocols.

For all patients a final follow-up visit is planned at D28 from randomization, regardless of the initial treatment assigned.

The following outcomes should be assessed:

- Hospitalization.
- ICU admission
- Hospital discharge
- Oxygen requirements.
    - Oxygen mask with reservoir bag.

CONFIDENTIAL

- Non-invasive mechanical ventilation (via mask) or high flow oxygen nasal tubes
- Mechanical ventilator requirement (via endotracheal tube or tracheostomy tub).
- ECMO requirement.

- Ordinal scale score:
  1. Not hospitalized, no limitations on activities.
  2. Not hospitalized, limitation on activities.
  3. Hospitalized, not requiring supplemental oxygen.
  4. Hospitalized, requiring supplemental oxygen.
  5. Hospitalized, on non-invasive ventilation or high flow oxygen devices or oxygen mask with reservoir bag.
  6. Hospitalized, on invasive mechanical ventilation or ECMO.
  7. Death.
- PaO2/FiO2 Index or SpO2/FiO2 Index.
- Temperature ºC
- Chest radiography (at least one during the follow-up period, recommended between D3-5, unless medically indicated any other day as part of the routine care)
- Laboratory disease parameters (CRP, d-dimer, IL-6, LDH, lymphocite counts, ferritine, plus any routing laboratory determinations usually performed by the patients´s physicians)
- Death.
- Concomitant medications
- Brescia-COVID score
- Adverse events: Adverse events (AEs), serious AEs, and laboratory abnormalities from the signature of the informed consent form or oral consent documentation, where appropirate. At inclusion they will be collected from the beginning of sarilumab administration. AEs will be documented as per the National Cancer Institute’s Common Terminology Criteria for Adverse Events (CTCAE) version 5.0 (Appendix 4).

If the patient has been discharged, they will be contacted by phone. If possible, a visit will be scheduled to complete the evaluation.

Follow-up period

At any time during the study, the patient may be seen in clinic for a visit outside of the protocol-specified visit schedule. Selected tests as required may be performed at the discretion of the Investigator Team. The frequency of clinical visits could increase if deemed necessary because of clinical situation, incapacity or demands of the patient. In the event that a study subject leaves the study early (i.e., early termination) and additional visit will be obtained with additional testing as indicated at the discretion of the Principal Investigator.

The Principal Investigator may decide to conduct final analysis of study data at any time, even while some subjects may remain in long-term follow-up. This decision may also take place after all

CONFIDENTIAL

study subjects have been completed the scheduled 28-day follow-up period for each patient. The timing of data analysis is the prerogative of the Principal Investigator, regardless of continuing long-term follow-up of study subjects.

CONFIDENTIAL

Table 2. SCHEDULE OF ACTIVITIES (SOA)

The parameters, clinical history, laboratory and instrumental data of patients will be collected according to the following scheme:

**Procedure**	**V0**	**V1**	**V2**	**V3**	**V4**	**V4 Follow-up**
**Day (+/Window**	**Screening**	**Baseline/ First dose**	**72h**	**5 day**	**15 day (+2)**	**28 day (+/-2)**
Informed Consent	X					
Inclusion/exclusion criteria	X	X				
Demography and medical history	X					
SARS-CoV-2 test	X					
COVID-19 history	X					
Rx Thorax		X[b]	X[a]	X[a]	X	
Randomization		X				
Study treatment administration		X				
Concomitant medication		X	X	X	X	
Laboratory tests		X[c]	X	X	X	
Optional blood for exploratory studies		X[d]				
Pregnancy Test	X					
PaO2/FiO2 Index		X	X	X	X	
Oxygen requirements, Oxygenation parameters (SaO2, arterial blood gases)		X	X	X	X	
Clinical data collection		X	X	X	X	X
Physical examination	X	X	X	X	X	
Laboratory tests	X[c]	X[c]	X	X	X	
Brescia-COVID score	X	X	X	X	X	
Vital signs: SpO2 /Tª		X	X	X	X	
Mechanical ventilator requirement		X	X	X	X	
Mortality			X	X	X	X
AE /SAE reporting		X	X	X	X	X

a- At least one radiographic assessment will be conducted during the follow-up visits, initially scheduled at D3 visit or D5 visit, unless otherwise medically indicated
b- Valid any imagin available up to 96h prior to randomisation
c- A complete laboratory parameters assessment should be available within 24 hours prior to randomisation
d-An additional blood sample will be collected and stored at HUPH biobank for future studies

CONFIDENTIAL

CONFIDENTIAL

Table 3. SCHEDULE OF ACTIVITIES OPEN-LABEL RESCUE PHASE (SOA) (SOC group without sarilumab progressing to severe respiratory failure)

The parameters, clinical history, laboratory and instrumental data of patients will be collected according to the following scheme:

Procedure	V1	V2	V3	V4 F/Uf	V5 follow up*
**Day (+/Window**	**Sarilumab rescue treatment**	**72h**	**5 day (±1)**	**15 day (+2)**	**28-day (+/-2) from initial randomisation**
Inclusion criteria (Patients in SOC group without sarilumab progressing to severe respiratory failure fulfilling as defined by the presence of BRESCIA-COVID 2-3 plus inflammatory markers)	X				
Rx Thorax	X			X	
Sarilumab administration	X				
Concomitant medication	X	X	X	X	
Laboratory disease parameters b	X	X	X	X	
PaO2/FiO2 Index	X	X	X	X	
Oxygen requirements, Oxygenation parameters (SaO2, arterial blood gases)	X	X	X	X	
Clinical data collection	X	X	X	X	X
Physical examination	X	X	X	X	
Brescia-COVID Scale	X	X	X	X	
Vital signs: SpO2 /Tª	X	X	X	X	
Mechanical ventilator requirement	X	X	X	X	
Mortality		X	X	X	X
AE /SAE reporting	X	X	X	X	X

CONFIDENTIAL

Study outcomes

Primary Endpoint

The primary endpoint is the proportion of patients progressing to severe respiratory failure (Brescia-COVID ≥2), ICU admission, or death.

Secondary endpoints

1. Time to progression to severe respiratory failure
2. Time to reduction of supplemental oxygen requirements
3. Time to non-invasive or invasive ventilation.
4. Proportion of patients with at least 1 point progression in the 7-point ordinal scale from randomization up to day 15

   Ordinal scale:

    1. Not hospitalized, no limitations on activities.
    2. Not hospitalized, limitation on activities.
    3. Hospitalized, not requiring supplemental oxygen.
    4. Hospitalized, requiring supplemental oxygen.
    5. Hospitalized, on non-invasive ventilation or high flow oxygen devices or oxygen mask with reservoir bag.
    6. Hospitalized, on invasive mechanical ventilation or ECMO.
    7. Death.
5. Rate of hospital discharge
6. Duration of hospitalization
7. Mortality rate and survival time.
8. Rate of ICU admission
9. Changes in serum levels of inflammatory cytokines.
10. Proportion of patients showing more than >1 organ failure, e.g. cardiovascular, liver and kidney failure
11. Safety and tolerability – Adverse event: The occurrence of treatment adverse events (AEs), serious AEs (SAEs), and laboratory abnormalities in the following, but not limited to, parameters: acute phase reactants CRP and ESR, complete blood count (including ANC and platelets), liver enzymes (ALT, AST and AP), and lipid levels, (time frame: up to day 15)

Calendar of Evaluation of Trial Variables

Safety will be evaluated through the collection of all AE for up to 28 days.

The Principal Investigator may decide to conduct interim analysis of study data at any time, even while some subjects may remain in follow-up. The timing of data analysis is the prerogative of the Principal Investigator, regardless of continuing long-term follow-up of study subjects.

9. PHARMACOVIGILANCE/ADVERSE EVENTS/SAFETY

All adverse events will be recorded in the CRF and graded according to the NCI-CTC v.5.0 (See Appendix 4).

Definitions

Adverse Event is defined as any event that results in worsening of the health of the subject of the clinical trial, regardless of relationship to the experimental therapy. It can be any symptom, sign, illness or experience, including abnormal results of diagnostic procedures, that develops or worsens in severity during the course of the study.

Any medical condition that is present at the time that the subject is screened will be considered as baseline and not reported as an AE. However, if the severity of any pre- existing medical condition increases, it should be recorded as an AE.

Given the nature of severity of the underlying illness, subjects will have many symptoms and abnormalities in vitals and laboratory. Only Grade 3 and 4 AEs will be captured as AEs in this trial, according to the following classification:

- Mild (Grade 1): Events that are usually transient and may require only minimal or no treatment or therapeutic intervention and generally do not interfere with the subject's usual activities of daily living.
- Moderate (Grade 2): Events that are usually alleviated with additional specific therapeutic intervention. The event interferes with usual activities of daily living, causing discomfort but poses no significant or permanent risk of harm to the research subject.
- Severe (Grade 3): Events interrupt usual activities of daily living, or significantly affects clinical status, or may require intensive therapeutic intervention. Severe events are usually incapacitating.
- Very severe (Grade 4): Events that are potentially life threatening.

Serious Adverse Event are defined as any AE (or adverse reaction) that in the view of either the investigator or the sponsor, it results in any of the following outcomes:

- Fatal
- Life-threatening*
- Requires or prolongs hospital stay
- Results in persistent or significant disability or incapacity
- A congenital anomaly or birth defect

The concept "life-threatening" means that in the opinion of the investigator, the patient is in real danger of death in the AE situation; it does not mean that the AE could have caused death if it would have been more intense.

Any AE that constitute an important medical event, will be treated as a SAE regarding notification procedures. Important medical events are those that may jeopardize the subject, and may require intervention to prevent one of the other serious outcomes noted above. All proven incidents of transmission of an infectious agent through the delivery of experimental therapy will also be

reported as SAEs.

Adverse reaction is any untoward medical occurrence associated to the administration of a research drug. It is different to an AE in that, in the AR, there is a suspicion of causal relationship between the research drug and the effect.

For each reported adverse event, the PI or designee must assess the relationship of the event to the study product using the following guideline:

- Related – The AE is known to occur with the study intervention, there is a reasonable possibility that the study intervention caused the AE, or there is a temporal relationship between the study intervention and event. Reasonable possibility means that there is evidence to suggest a causal relationship between the study intervention and the AE.
- *Not Related* – There is not a reasonable possibility that the administration of the study intervention caused the event, there is no temporal relationship between the study intervention and event onset, or an alternate aetiology has been established.

The relationship or causality of the AE to sarilumab therapy will be determined by the PI of the center, or the person in whom she/he delegates.

Suspected Unexpected Serious Adverse Reaction (SUSAR)

A SUSAR is a serious adverse reaction (SAR) as noted above, that is also unexpected. Unexpectedness is defined as an adverse event in which the nature, severity, specific or consequences have not been previously observed or noted in the reference information for the drug or that has been noted in the same drug class. An ADR with a fatal outcome is considered unexpected.

Registry of AE

All AE will be documented, including the ones observed during clinical visits and the ones reported by the patient. AEs will be documented throughout the duration of the trial. The patient should be asked about possible AEs in the time between clinical visits.

All AE will be documented in the CRF and in the medical records.

All AE will be followed until resolution, until 15 days after the administration of the first dose of sarilumab (V1), or until toxicity is considered non-reversible or is grade 1.

SAE that do not require expedited notification

The patients who will participate in this study have a poor prognosis, with the possibility of death due to the disease. This possible progression is included in the primary endpoint, so it will not be necessary to report deaths as SAE in this study.

This event will be documented in the medical history and in the CRF of the patient. The rest of the SAE will be reported by the investigator following the expedited report procedure as follows.

Procedure of notification

Reporting of SAEs

In case of occurrence of a SAE that must be reported to pharmacovigilance, a SAE Serious Adverse Events Reporting Form must be completed and signed by the investigator within 24 hours. The investigator will keep a copy of this SAE form on file at the study site.

When additional information about the SAE becomes available, or when the SAE is resolved, or when any change is unexpected, the investigator must provide further information in the form of a written narrative that should also be sent to pharmacovigilance. The responsible will review the received form and will ask for additional information if needed. In the event of SUSAR, follow-up information will be provided.

SAE appearing more than 15 days from the end of the experimental therapy will be reported if the investigator considers there is a causal relationship to the therapy or if it is of medical importance.

Notification of Laboratory Abnormalities

All laboratory results must be filed in the subject's medical record and be monitored. The investigator must review laboratory results in a timely manner demonstrated by signature/date and assignment of clinical significance assessment. Laboratory abnormalities that are not clinically significant, i.e., minor deviations from the normal range, are expected and it is likely that no medical intervention will be required. Such results will not be considered to be adverse events.

Clinically Significant (CS) Laboratory Abnormalities

All CTCAE Grade 3 and 4 laboratory result abnormalities and any laboratory abnormality that is considered to be clinically significant by the investigator will be recorded on the AE case report form. An abnormal test result will be considered an AE if:

- It is not associated with an already reported AE, diagnosis or pre- existing condition
- There is a change in concomitant medication or intervention as needed, in direct response to the Grade 3 or 4 laboratory result
- The investigator exercises his/her discretion to make significance determinations for any subject laboratory result
- All such laboratory abnormalities will be repeated and assessed as soon as possible for "seriousness" by the investigator or licensed physician, and if they meet the regulatory definition of "serious", they will be reported as SAEs following regulatory and protocol requirements. Repeat laboratory tests may be run in order to monitor the result.

Serious Laboratory Abnormalities

Any laboratory abnormality meeting the regulatory definition of "serious" must be recorded both on the adverse event case report form and the Serious Adverse Event Form. If a subject experiences a serious toxicity or dies, the local and national required regulatory authorities will be notified within 24 hours of awareness of the event, as required.

Urgent or expedited notification of SUSAR to health authorities

Responsible Pharmacovigilance person will notify all SUSAR to the AEMPS following the procedures approved by law. Any SUSAR will be reported within 15 calendar days from the moment when the sponsor has knowledge of it. Any fatal or life-threatening, SUSAR, should be reported within 7 days of the moment the sponsor had knowledge of it. The initial information should be completed, if possible, within 8 additional days.

Urgent notification of other relevant safety information

Urgent notification to AEMPS (Área de ensayos clínicos de la Subdirección Gral. De Medicamentos de Uso Humano), will also be employed for all the information that could modify the risk-to-

benefit balance of the experimental therapy or advice changes in the therapy, or in the trial.

Regulatory authorities will be notified of the new information will be notified as soon as possible and no later than 15 days from when the investigator has knowledge of it. Additional information will be provided also as soon as possible.

Annual safety report

All SUSAR and SAEs reported will be notified to the AEMPS and the CEIm in an annual safety report.

10. STATISTICAL ANALYSIS

The statistical analysis will be carried out in accordance with the principles specified in the International Conference on Harmonization (ICH) Topic E9 (CPMP / ICH / 363/96)1. A detailed Statistical Analysis Plan (SAP) agreed upon by the CT Executive Board and the Project Statistician will be available early during the recruitment phase. This SAP will follow the general regulatory recommendations given in the ICHE9 guidance, as well as other specific guidance on methodological and statistical issues2. Also, it will stick to the recommendations given by the consensus documents of the scientific journals3,4 to improve reliability and value of medical research literature by promoting transparent and accurate reporting of clinical research studies.

The SAS System5 (Release 9.4, or an upgraded version), or equivalent validated statistical software, will be the statistical software used to analyse the data sets.

A summary of the overall approach to statistical analysis is presented hereafter.

Sample size estimation

To obtain a power of 80.00% to detect differences in the contrast of the null hypothesis H_0: p1 = p2 by means of a bilateral Chi square test for two independent samples, taking into account that the level of significance is 5.00%, and assuming that the proportion in the Reference group is 25.00%, the proportion in the Experimental group is 10.00%, and that the proportion of experimental units in the Reference group with respect to the total is 50.00 % it will be necessary to include 100 experimental units in the Reference group and 100 units in the Experimental group, totalling 200 experimental units in the study.

Data Review (DR)

The Data Review (DR) will be performed before lock of database. Data will be examined for compliance with the trial protocol by the monitor and the data manager. Deviations will be sent to the project statistician to plan listings for the Data Review (DR). The objective is to carry out the population selection and definition of the final study populations as well as a preliminary assessment of the quality of the trial data.

---

[1] CPMP/ICH/363/96. ICH E9 Statistical Principles for Clinical Trials. URL: http://www.ema.europa.eu/ema/pages/includes/document/open_document.jsp?webContentId=WC500002928, last access: 20-Mar-2020.

[2] EMEA Scientific Guidelines for Human Medicinal Products, Clinical Efficacy and Safety Guidelines, General Guidelines. URL: https://www.ema.europa.eu/en/human-regulatory/research-development/scientific-guidelines/clinical-efficacy-safety/biostatistics , last access: 20-Mar-2020.

3 Schulz KF, Altman GD, Moher D for the CONSORT Group*. CONSORT 2010 Statement: Updated Guidelines for Reporting Parallel Group Randomized Trials. Ann Intern Med. 2010;152:726-732.

4 EQUATOR-network (Enhancing the Quality and Transparency of Health Research). URL: http://www.equator-network.org/resource-centre/library-of-health-research-reporting/, last access 20-Mar-2020

[5] SAS version 9.4 software, SAS Institute Inc., Cary, NC, URL: http://www.sas.com/, last access: 20-Mar-2020

Analysis populations

There will the following analysis populations for this study:

1. Full Analysis Set (FAS): All patients who are randomized into the study will be included in the FAS population.
2. Per Protocol Population: Per protocol (PP) patient sets will be defined as those patients included in the FAS set without major protocol deviations that might impact the study's main assessments. These deviations will be assessed during the data review prior to database lock.
3. The Safety population is defined as all randomized participants who received the investigational product.

The precise reasons for excluding participants from each population will be fully defined and documented independently of the randomization codes during the data blinder review and before data lock.

Randomisation Procedure

Randomisation codes were produced by means of the PROC PLAN of the SAS system, with a 1:1 ratio of assignment between both arms, stratifying by centre, blocks multiple of 2 elements. The randomisation schedule will be managed from the eCRF in a concealed manner.

Statistical analysis

Descriptive analysis

A descriptive analysis will be performed for all parameters overall and by arm at every study time-point. Categorical parameters will be presented by means of frequencies and percentages. Continuous parameters will be summarized by means of the appropriate descriptive statistics (mean ± standard deviation or median and interquartile range).

The efficacy and safety endpoints will be descriptively compared between study arms. Changes from baseline, when applicable, will also be summarized by study arm.

Inferential analysis

The inferential analyses will be limited to the efficacy variables, and the adverse events.

Primary endpoint

The primary endpoint is the proportion of patients progressing to severe respiratory failure (Brescia-COVID ≥2), ICU admission, or death. The proportion of patients with failure at day 7, will be estimated using a log-binomial regression model including stratification variables. In the unexpected event that the model does not fit, the Poisson regression model with long-link and robust variance estimator will be used instead.

Secondary endpoints and safety outcomes

Binary outcomes

Binary efficacy and safety outcomes will be analysed as described for the primary endpoint.

Shift outcomes

The median of the absolute values the 95% confidence interval (95%CI) will calculated using the Hodges-Lehmann methods (i.e. median of all cross differences between treatments based on the Mann-Whitney distribution).

Continuous outcomes

Continuous variables will be analysed using Mixed Models, including in the model the baseline measurement, the stratification variables, treatment as well as the interaction between treatment and time, declaring time as categorical. The variance-covariance matrix will be fixed initially as unstructured. Contrasts between study groups will be performed by time-point. The treatment effect will be estimated through adjusted means –Least Square Means (LSMeans) – its standard error – Standard Error of Mean (SEM)- and its 95%CI. Differences between treatments will be estimated through the differences between LSMeans, SEM and 95%CI.

Survival endpoints

The survival function as well as the median [95% confidence interval -95%CI-] time to event will be estimated by means of the Kaplan-Meier method. Group comparisons will be done using the (stratified) log-rank test and the (stratified) hazard ratios -HR- (95%CI) were taken from the Cox model.

General strategy for the rest of variables

The rest of variables will be analysed according to the following strategy: The Fisher's exact test used for categorical variables, the t-test for Gaussian distributed variables and, for non-Gaussian continuous or ordinal variables, non-parametric methods (Mann-Whitney test).

Criteria for ending the trial

The study will end when all patients have been recruited, the last patient has completed the study procedures, and all queries and AEs have been solved. The sponsor can terminate prematurely the study.

The reasons to terminate prematurely or suspend a trial include, but are not limited to:

-Finding of unforeseen risks for the patients that are considered unacceptable -There is evident that the recruitment of patients is not adequate in number or quality.

-Incorrect data collection

-AE rate or severity points to a safety risk

In all the previous situations, a final evaluation of the patients included in the study should be made, and the CRF will be completed, as much as possible.

Procedures to communicate deviations from planned statistical analysis.

All deviations from the planned analysis would be reflected in the final report of the study.

Subject included in each analysis

There will the following analysis populations for this study:

1. Full Analysis Set (FAS): All patients who are randomized into the study will be included in the FAS population.
2. Per Protocol Population: Per protocol (PP) patient sets will be defined as those patients

included in the FAS set without major protocol deviations that might impact the study's main assessments. These deviations will be assessed during the data review prior to database lock.

3. The Safety population is defined as all randomized participants who received the investigational product.

The precise reasons for excluding participants from each population will be fully defined and documented independently of the randomization codes during the data blinder review and before data lock.

Missing data

The handling of missing data will follow the principles specified in the ICH-E9 and the CPMP/EWP/1776/99 Rev1. Guideline on Missing Data in confirmatory trials Guidelines. In principle, the rate of missing data is estimated to be very low due to the type of endpoint, easily available with a fast-clinical assessment, so no impact is expected in the primary analysis. In any case, a very conservative strategy will be implemented consisting of imputing any missing data or other binary efficacy secondary outcomes will be considered to failures, irrespectively to the reason for missingness.

11. ETHICAL ASPECTS

Good Clinical Practice

This study will be conducted in full conformance with the ICH E6 guideline for Good Clinical Practice and the principles of the Declaration of Helsinki, or the local laws and regulations.

The investigator will be familiar with the use of the experimental therapy as described in the protocol.

Essential clinical documents will be kept to prove validity and integrity of data. Essential files of the trial will be defined at the beginning of the trial, and kept during and after it, following current laws.

Ethical considerations

A Research Ethics Committee (REC) will review and approve this protocol, associated informed consent documents, prior to the recruitment, screening, and enrolment of subjects.

Any amendments to the protocol or consent materials will be approved by the REC before they are implemented.

Information to the subjects and informed consent

Informed consent is a process that is initiated prior to an individual agreeing to participate in a trial and continuing throughout the individual's trial participation. Investigators will obtain the subject's informed consent in accordance with the RD1090/2015, *4 de diciembre, por el que se regulan los Ensayos Clínicos con Medicamentos*, and the internationally accepted guidance.

Subjects will receive a concise and focused presentation of key information about the clinical trial. Due to paper handling limitation in COVID wards, oral witnessed consent will be accepted before entering into the trial. Written consent form will be obtained from the patient himself or acceptable representatives whenever possible. The key information about the study will be

organized and presented in lay terminology and language that facilitates understanding why one might or might not want to participate.

Confidentiality of the patients

Participants will be assigned a unique identifier. Any participant records or datasets that are introduced in the eCRF or transferred outside the clinical site will contain the identifier only; participant names or any information which would make the participant identifiable will not be transferred.

The participant must be informed that his/her medical records may be examined by monitors, Clinical Quality Assurance auditors or other authorized personnel appointed by the sponsor, by appropriate CEIm members, and by inspectors from regulatory authorities.

The data must be collected and processed with adequate precautions to ensure confidentiality and compliance with applicable data privacy protection laws and regulations (Reglamento (UE) 2016/679 and Ley orgánica 3/2008).

Financial responsibility

Trial medication will be provided by the sponsor who has obtained an agreement with SANOFI to obtain free of charge medication supply. The sponsor will not finance the procedures that would be done as standard of care nor other medical needs of the patient, not related to the trial.

Insurance

This is a low intervention clinical trial and therefore there is no need to specific insurance covering legal responsibility for trial- related injuries. According to RD 1090/2015, any damage to study subjets suffered as a result of the trial will be covered by the professional civil liability insurance or equivalent financial guarantee of the site where the clinical trial is conducted.Notification to health authorities

This trial needs a request to Agencia Española de Medicamentos y Productos Sanitarios (AEMPS) for approval. The trial need the approval before starting.

Adherence to protocol

The investigator will conduct the trial according to the protocol, after the approval of competent authorities and CEIm.

Protocol will not be modified without approval of sponsor and competent authorities. All protocol amendments require previous approval of CEIm with the exception of situations where the change is necessary to avoid immediate risk for the patient.

If any change is needed to avoid imminent risk to the patients, the investigator will contact the Sponsor to propose the changes. Any deviation to the protocol should be documented and the original records.

Premature termination of the trial

This study may be prematurely terminated if there is sufficient reasonable cause, including but not limited to:

- Determination of unexpected, significant, or unacceptable risk to subjects
- Results of interim analysis

- Insufficient compliance to protocol requirements
- Data that are not sufficiently complete and/or not evaluable
- Regulatory authorities

If the study is prematurely terminated, the site PI will inform study subjects and the REC as applicable.

The sponsor will notify regulatory authorities as applicable

12. PRACTICAL CONSIDERATIONS

Direct access to data/ source documents

The principal investigator and the sponsor will guarantee direct access to all data and source documents as needed for monitoring of the trial as well as audit and review by CEIm and Health Authorities.

Responsibilities of all participants.

Principal investigator

Must agree with the present protocol and have a deep knowledge of the characteristics of the products used in the trial.

The investigator should present the patient information sheet to the patients and help them to understand the explanation given. Is important to explain to the patient that the participation is voluntary, that his/her decision will not affect to the relationship between patient and doctor and that all person involved in the study will respect the confidentiality of the information.

The principal investigator his/her co-worker will collect, document and properly report the data. The principal investigator will be responsible of the urgent reporting of SAEs and SUSARs within 24 h.

Principal investigator will inform the CEIm about the evolution and will cooperate with the sponsor in the final report.

Monitor

Clinical site monitoring is conducted to ensure that the rights and well-being of trial subjects are protected, that the reported trial data are accurate, complete, and verifiable. Clinical Monitoring also ensures conduct of the trial is in compliance with the currently approved protocol/ amendment(s), ICH, GCP, and with applicable regulatory requirement(s) and sponsor requirements.

Monitoring visits will include, but are not limited to, review of regulatory files, accountability records, CRFs, ICFs, medical and laboratory reports, site study intervention storage records, training records, and protocol and GCP compliance.

The monitoring visits can be made online.

The investigator will permit study-related monitoring, audits, and inspections by the CEIm, the sponsor, government regulatory bodies, and quality assurance groups of all study related documents.

Sponsor

The Sponsor is Cristina Avendaño Sola, MD, PhD, Medical Staff at Clinical Pharmacology Department, Hospital Universitario Puerta de Hierro. The Sponsor is responsible for the initiation, management and termination of the trial.

The sponsor is responsible for securing agreement from all involved parties to ensure direct access to all trial-related sites, source data/documents, and reports for the purpose of monitoring and auditing by the sponsor, and inspection by domestic and foreign regulatory authorities.

The sponsor is responsible to ensure that trial is conducted and data are generated, documented (recorded), and reported in compliance with the protocol, GCP, and the applicable regulatory requirement(s).

Audit

The investigator will permit study-related monitoring, audits, and inspections by the CEIm, the sponsor, government regulatory bodies, and quality assurance groups of all study related documents (e.g. source documents, regulatory documents, data collection instruments, study data, etc.). The investigator will ensure the capability for inspections of applicable study-related facilities (e.g. pharmacy, diagnostic laboratory, etc.).

Use and publication or study results

The study results may be published in scientific journals, or presented at scientific meetings by the investigator following agreement of the Sponsor with Sanofi, the manufacturer and provider of Sarilumab, with mention of the CEIm that approved the study.

The study results will be subject to the terms agreed by the Sponsor and Sanofi in the Investigator Initiated Research Agreement signed in relation to this study.

13. FILE KEEPING

Case Report Form (CRF)

Will be filled on the electronic clinical trial platform by the investigator team from the original data source of the clinical record of the patients.

Documents in custody of the investigator:

The investigator will keep all the original documents of the clinical records for each patient and a list of all the patients included for 25 years or until an agreement with the sponsor. This documentation will not be destroyed without the sponsor agreement. The investigator will custody all the files in agreement with the International conference on Harmonisation (ICH)

Trial Master File

The investigator and institution will maintain the essential trial documents in the Trial Master File (TMF) as specified in the GCP guidelines (ICH:E6: Section 8.0) and as required by the applicable regulatory requirement(s). The investigator/institution should take measures to prevent accidental or premature destruction of these documents.

Monitoring

The sponsor will assign monitoring personal to the trial. His roles will include helping the investigator and the sponsor to keep data well organized and easy to access.

Final Report

The investigator team will make a final report to be presented to the Health authorities.

14. LITERATURE REFERENCES

1) Geng Li1, Coronavirus infections and immune responses. J Med Virol. 2020;92:424–432.

2) Hasan K. Siddiqi MD, MSCR , Mandeep R. Mehra MD, MSc , COVID-19 Illness in Native and Immunosuppressed States: A Clinical-Therapeutic Staging Proposal, Journal of Heart and Lung Transplantation (2020), doi: https://doi.org/10.1016/j.healun.2020.03.012

3) Xiaoling Xu et al. Effective Treatment of Severe COVID-19 Patients with Tocilizumab. Disponible en: https://www.ser.es/wp-content/uploads/2020/03/TCZ-and-COVID-19.pdf)

4) Tratamientos disponibles para el manejo de la infección respiratoria por SARS-CoV-2. AEMPS link: https://www.aemps.gob.es/la-aemps/ultima-informacion-de-la-aemps-acerca-del-covid%e2%80%9119/tratamientos-disponibles-para-el-manejo-de-la-infeccion-respiratoria-por-sars-cov-2/

5) Acute Respiratory Distress Syndrome Advances in Diagnosis and Treatment. JAMA. 2018;319(7):698-710. doi:10.1001/jama.2017.21907

6) Wu C, Chen X, Cai Y, et al. Risk Factors Associated With Acute Respiratory Distress Syndrome and Death in Patients With Coronavirus Disease 2019 Pneumonia in Wuhan, China [published online ahead of print, 2020 Mar 13]. JAMA Intern Med. 2020;e200994.

7) Ruan Q, Yang K, Wang W, Jiang L, Song J. Clinical predictors of mortality due to COVID-19 based on an analysis of data of 150 patients from Wuhan, China [published online ahead of print, 2020 Mar 3]. Intensive Care Med. 2020;10.1007/s00134-020-05991-x

8) Zhou F, Yu T, Du R, et al. Clinical course and risk factors for mortality of adult inpatients with COVID-19 in Wuhan, China: a retrospective cohort study [published online ahead of print, 2020 Mar 11] [published correction appears in Lancet. 2020 Mar 12;]. Lancet. 2020;S0140-6736(20)30566-3.

9) Tanaka T, Narazaki M, Kishimoto T. Immunotherapeutic implications of IL-6 blockade for cytokine storm. Immunotherapy. 2016;8(8):959–970.

APPENDIX 1: RESEARCH TEAM

Hospital Universitario Puerta de Hierro Majadahonda

Principal Investigator:

Ana Fernández Cruz, MD, PhD. Department of Internal Medicine. Infectious Diseases Unit.

Co-Investigators:

Elena Muñez, MD, PhD. Department of Internal Medicine. Infectious Diseases Unit.

Antonio Ramos MD, PhD. Department of Internal Medicine. Infectious Diseases Unit.

Pablo Tutor MD, PhD. Department of Internal Medicine.

Susana Mellor MD, PhD. Department of Internal Medicine.

Víctor Moreno-Torres MD. Department of Internal Medicine.

Alejandro Callejas, MD. Department of Internal Medicine.

Isolina Baños, MD, PhD. Department of Internal Medicine

Jesús Sanz Sanz, MD. Department of Rheumatology

Elena Diago Sempere, MD. Department of Clinical Pharmacology

Isabel Salcedo de Diego, BSN, PhD. Department of Clinical Pharmacology

Appendix 2: INFORMED CONSENT/ INFORMATION SHEET

Attached form.

APPENDIX 3: INSURANCE CERTIFICATE

A request to clasiffy the study as a low intervention trial to the REC at Hospital Puerta de Hierro has been sought. The need for an insurance certificate is subjected to the outcome of the former decision

APPENDIX 4: NCI-CTC AE-CRITERIA

Common terminology criteria for classification of adverse events version 5.0 (NCI CTC AEv5) are available at the following Internet address:

https://ctep.cancer.gov/protocolDevelopment/electronic_applications/ctc.htm

APPENDIX 5: SmPC Kevzara

https://cima.aemps.es/cima/dochtml/ft/1171196003/FT_1171196003.html

APPENDIX 6: Brescia-COVID Scale

APPENDIX

Brescia-COVID respiratory severity scale	
0	Ambient air
1	Oxygen therapy
2	Oxygen therapy plus 1 of the following criteria:<br>a) The patient has dyspnoea or **STACCATO SPEECH** (inability to count rapidly to 20 after taking a deep breath) at rest or after minimum activity (sitting down on bed, standing up, speaking, swallowing, coughing)<br>b) Respiratory rate > 22 with >6L/minute O2<br>c) PaO2 <65mmHg with >6L/minute O2<br>d) Significant worsening of chest x-ray (increased compactness and extension of infiltrate)
3	**The patient requires high-frequency nasal ventilation (HFNC), CPAP or NIV**
4	The patient is intubated for **CPAP** or pressure support
5	The patient is under controlled mechanical ventilation; PaO2/FiO2 >150 mmHg
6	The patient is under controlled mechanical ventilation; PaO2/FiO2 ≤150 mmHg
7	The patient is under controlled mechanical ventilation; PaO2/FiO2 ≤150 mmHg ***and*** intravenous infusion of neuromuscular blockers
8	The patient is under controlled mechanical ventilation; PaO2/FiO2 ≤150 mmHg ***and*** one of the following:<br>a) Prone position<br>b) ECMO

https://www.regione.lazio.it/binary/rl_sanita/tbl_contenuti/COVID19_LG_SIMITLombardia_2.0.pdf

APPENDIX 7: List of Participating Centers and Principal Investigators

- Hospital Universitario Puerta de Hierro Majadahonda, Madrid
    - Principal Investigator: Ana Fernández Cruz, MD, PhD. Infectious diseases Unit. Internal Medicine Department
- Hospital H. Universitario Son Llàtzer de Palma de Mallorca
    - Principal Investigator: Juan Sebastian Buades Mateu, MD. Internal Medicine Department
- Hospital Ruber Juan Bravo, Madrid
    - Principal Investigator: Marta Sanz García, MD, PhD. Internal Medicine Department
- Hospital Universitario Infanta Sofia, Madrid
    - Principal Investigator: Eduardo Malmierca, MD, PhD. Internal Medicine Department
- Complejo Asistencial de Segovia, Segovia
    - Principal Investigator: Marta Gonzalez Rozas, MD, PhD. Internal Medicine Department
